# Training Genetic Counsellors to Deliver an Innovative Therapeutic Intervention: their Views and Experience of Facilitating Multi-Family Discussion Groups

**DOI:** 10.1007/s10897-016-0008-0

**Published:** 2016-10-10

**Authors:** Ivan Eisler, Frances Flinter, Jo Grey, Suzanne Hutchison, Carole Jackson, Louise Longworth, Rhona MacLeod, Marion McAllister, Alison Metcalfe, Christine Patch, Buddug Cope, Glenn Robert, Emma Rowland, Fiona Ulph

**Affiliations:** 1 South London & Maudsley NHS Foundation Trust, London, UK; 2 Guy’s and St Thomas’ NHS Foundation Trust, London, UK; 3 Association for Multiple Endocrine Neoplasia Disorders (AMEND), London, UK; 4 Kings College London, London, UK; 5 Brunel University London, London, UK; 6 Central Manchester University Hospitals NHS Foundation Trust, London, UK; 7 Cardiff University, Cardiff, UK; 8 Genetic Alliance UK, London, UK; 9 University of Manchester, Manchester, UK

**Keywords:** Multi-family discussion groups, Genetic counsellors, Family communication, Therapeutic intervention, Genomics, Inherited genetic conditions

## Abstract

Innovations in clinical genetics have increased diagnosis, treatment and prognosis of inherited genetic conditions (IGCs). This has led to an increased number of families seeking genetic testing and / or genetic counselling and increased the clinical load for genetic counsellors (GCs). Keeping pace with biomedical discoveries, interventions are required to support families to understand, communicate and cope with their Inherited Genetic Condition. The Socio-Psychological Research in Genomics (SPRinG) collaborative have developed a new intervention, based on multi-family discussion groups (MFDGs), to support families affected by IGCs and train GCs in its delivery. A potential challenge to implementing the intervention was whether GCs were willing and able to undergo the training to deliver the MFDG. In analysing three multi-perspective interviews with GCs, this paper evaluates the training received. Findings suggests that MFDGs are a potential valuable resource in supporting families to communicate genetic risk information and can enhance family function and emotional well-being. Furthermore, we demonstrate that it is feasible to train GCs in the delivery of the intervention and that it has the potential to be integrated into clinical practice. Its longer term implementation into routine clinical practice however relies on changes in both organisation of clinical genetics services and genetic counsellors’ professional development.

## Introduction

Innovations in genetics and genomics via the human genome project have increased the possibilities for diagnosis, treatment and prognosis of inherited genetic conditions (IGCs) (Middleton et al. 2015). These biomedical innovations and discoveries have led to an increased number of families seeking genetic testing and / or genetic counselling (Wang et al. 2004). This has subsequently impacted on the clinical load of genetic counsellors (GCs) and other health professionals (Bowles Biesecker and Marteau 1999; Sahhar et al. 2005). As the number of families affected or at risk from IGCs rises, there are increasing demands on genetic counselling services to support families in understanding and coping with their condition (Mendes et al. 2010; Wang et al. 2004). To keep pace with biomedical discoveries and resulting demands, GCS will need to adapt and evolve (Battista et al. 2012; Haspel et al. 2010; Sahhar et al. 2005; Weil 2002).

Currently the role of GCs is to provide both genetics education in a meaningful way to help the individual or family to understand the medical facts, the hereditary nature and risks of the condition and to provide emotional and psychological support for families affected or at risk from IGCs so that they may adjust to the condition (Biesecker and Peters 2001; Frazer 1974). Until recently, the 1975 American Society of Human Genetics (ASHG) definition of genetic counselling has been the most cited in the literature *(Begleiter* 2002 *:558)* (ASGH 1975)*.* However, in 2003 the National Society of Genetic Counsellors (NSGC) appointed a task force to asses and develop a new definition of genetic counselling to incorporate innovations in genomic medicine that move beyond traditional genetic counselling settings and into laboratory genetic medicine, public health, social and behavioural research and common diseases. The NSGC definition is provided below:

“Genetic counselling is the process of helping people understand and adapt to the medical, psychological and familial implications of genetic contributions to disease. The process integrates the following: *Interpretation of family and medical histories to assess the chance of disease occurrence and recurrence; Education about inheritance, testing, management, prevention, resources and research; Counselling to promote informed choices and adaption to the risk or condition (Resta et al.* 2006 *)*

Within this definition genetic conditions are recognised as family conditions (Mendes et al. 2010) and therefore the delivery of genetic risk information is not just an individual matter but a family concern (Mendes et al. 2010; McDaniel 2005; Peterson 2005; Rantanen et al. 2008; Rolland and Williams 2005). Genetic counsellors therefore advocate that affected or at risk family members communicate genetic risk information to their families, especially to their children (Elwyn et al. 2000; Rowland and Metcalfe 2013; Ulph et al. 2014). Whilst affected or at risk parents want to disclose information, they often find this task challenging due to the perceived negative impacts that this information will bring to their social and emotional lives (McAllister et al. 2007).

Families therefore require support from GCs in communicating genetic risk information and to support their psychosocial and emotional well-being (Michie et al. 1997) and have an expectation that health professionals will support them when needed (Ulph et al. 2014). However, despite the need for emotional support, Bosk (1992) found that genetic counselling services are ill-equipped to support families emotionally (Bosk 1992; Michie et al. 1997). This is often due to increasing patient volumes and limited human resources, which prevent GCs from following up patients. Genetic counsellors also receive limited training in interpersonal counselling and psychosocial assessment skills which could better assist them in supporting families emotionally (Begleiter 2002).

Without emotional and informational support from GCs, families may become overwhelmed with their genetic condition and experience disruption in crucial aspects of their family functioning as the genetic condition shapes their family identity (Mendes et al. 2015; Metcalfe et al. 2011; Metcalfe et al. 2008; Patterson and Garwick 1994; Sobel and Cowan 2003). To better support families’ emotional and psychological well-being, it is crucial that psychosocial, family-based interventions informed by theory are integrated within standard genetic counselling services (Mendes et al. 2010; Mendes et al. 2015; Metcalfe et al. 2008; Peterson 2005). Such interventions attempt to help the whole family by supporting them in adapting to, normalising and integrating risk management strategies into their lives (Goldbeck and Babka 2001; Mendes et al. 2015; Mendes et al. 2012).

Recent decades have seen a growth in the range of interventions, such as the Multi-Family Discussion Group (MFDG), which provide a combination of informational and psychological support for families (Asen 2002; Asen and Scholz 2010). MFDGs have been successfully integrated into a variety of health care services such as mental health (Asen and Schuff 2006; Eisler 2005; Lemmens et al. 2007; Satin et al. 1989; Wolpert et al. 2015) management of chronic disease (Gonzalez and Steinglass 2004; Kazak et al. 1999; Lemmens et al. 2005; McFarlane 2004; McKay et al. 2011), familial cancer (Mendes et al. 2015) and behavioural problems (Asen 2007). There are however few psychosocial, family-based interventions that support families through the complexities of dealing with genetic conditions (Chiquelho et al. 2011; Roberts et al. 2002; Steinglass 1998; Todd et al. 2002).

MFDGs are integrative interventions that draw on a range of concepts including systemic therapy, cognitive behaviour therapy and group therapy and practices and involve working with between 6 and 10 families at the same time. Families who share an experience are brought together and facilitated by therapists, to explore the issues they face and identify their family’s strengths in dealing with those issues (Asen and Scholz 2010). This builds the family’s sense of identity and self-esteem, reduces a sense of isolation and stigmatisation and assists them in building and maintaining strong supportive relationships (Asen and Scholz 2010).

MFDGs are based on the premise that families affected by the condition are better suited to understanding and making suggestions to other families about how to cope and adapt (Asen and Scholz 2010). In MFDG settings, trained facilitators help families to share their experiences of living with their condition and find new and more effective ways of managing it through facilitating discussions, group activities and experiential or creative exercises. Research has shown that both clinical and cost effectiveness are often improved when psychological interventions involve the whole family rather than just the patient (Crane and Christenson 2008; Kaslow et al. 2012), particularly when multiple families are present (Rolland and Williams 2005). MFDGs offer a safe context in which families can learn from and support each other (McFarlane 2004; Mendes et al. 2010; Ostroff et al. 2004) and therefore reduce families’ sense of isolation and stigma, increase coping and adaptability to the illness / condition, increase treatment adherence (Mendes et al. 2010) and help improve communication not only within families but between families and clinical staff (Asen and Scholz 2010; Mendes et al. 2010).

### Purpose of the Study

Our study involved agreeing on the aims and co-designing the content of a novel MFDG for IGCs with parents, children, young people and GCs (SPRinG collaborative 2015). Whilst we could have employed a family therapist to facilitate the intervention, we wanted to explore whether GCs could be trained to deliver the intervention. GCs have the knowledge about genetic conditions and have skills and experience in assisting people to understand risk information and make informed decisions. By contrast the family therapist has expertise in facilitating family communication, assisting families to adapt to new contexts and finding their own strengths and resources to cope with difficult or challenging situations. Our expectation was that by combining the two roles, it would be possible to assist families in communicating about the genetic condition more effectively and assist them in adapting to living with the IGC. Therefore we decided to see whether it was possible to train three GCs in MFDG facilitation techniques to improve family communication and coping with the IGC. This paper describes the GCs’ experiences and reflections as they participated in the process from the design of the intervention through to co-facilitating a full MFDG programme.

## Method

### Design

**Fig. 1:**
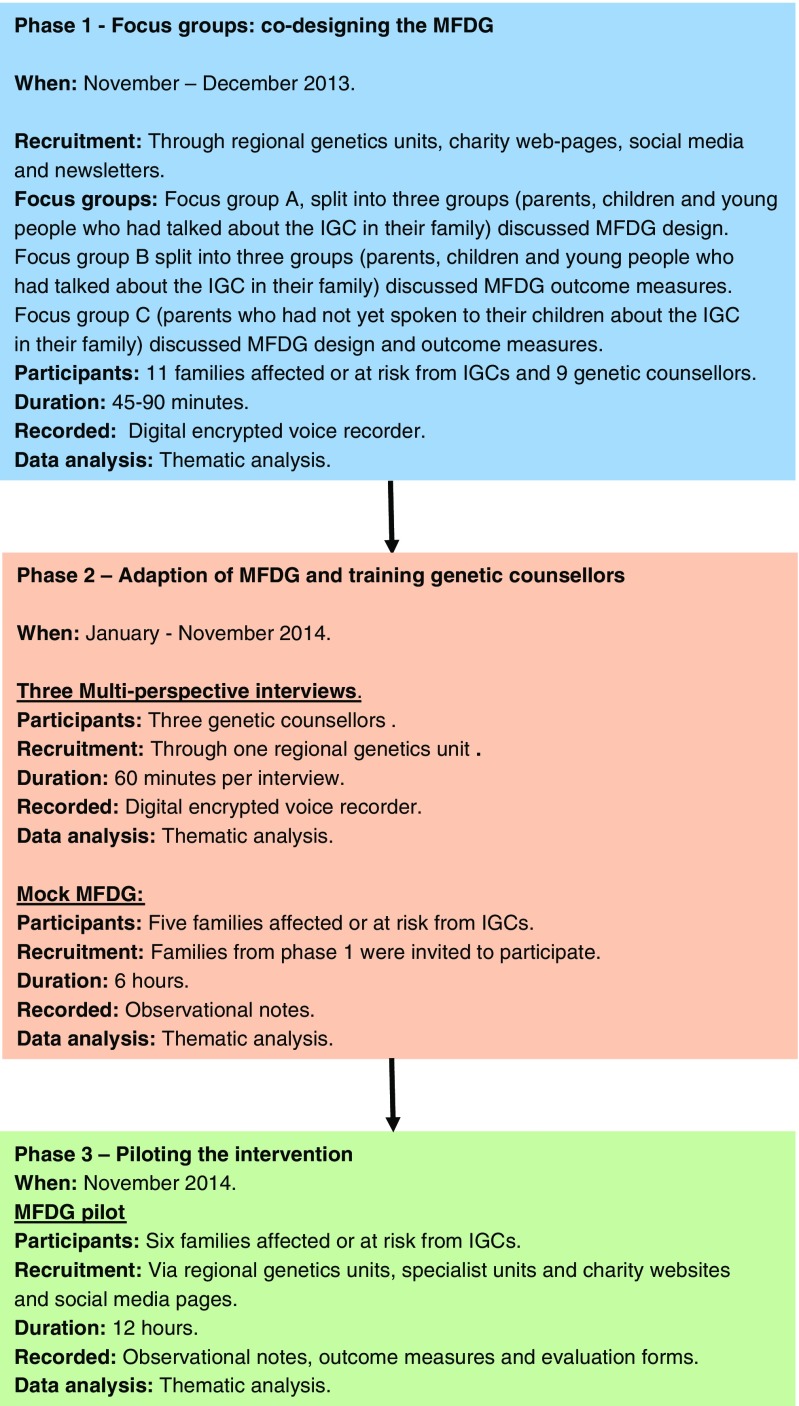
Diagram showing the three phases of the research project

Our research was conducted over three methodological phases: Phase 1 – focus groups to support the co-design of the MFDG intervention; Phase 2 - Adapting the MFDG intervention for use with families affected or at risk from IGCs and training GCs in its use; and Phase 3 – piloting the intervention (see Fig. 1). Phase 2 began in January 2014 and Phase 3 ended in November 2014, a period of 11 months. The full study has been published (SPRinG collaborative 2015) but here we undertake a separate analysis focusing on the GCs experiences and views of the MFDG, over the duration of their involvement. Ethical approval was given by NHS Riverside Ethics Committee, London Ref. 13/LO/0236.

### Procedure

Three GCs (Table 1) from a London genetics centre were trained in the MFDG intervention by two family therapists (SH and IE) and the Principle Investigator (AM). In the UK, clinical genetics services are provided free at the point of care by the National Health Service (NHS). The MFDG was adapted for use with families with IGCs following focus groups to support the design and development of the MFDG (SPRinG collaborative 2015). The GCs’ training included the receipt of a training manual containing literature and research pertaining to the intervention and how MFDGs are used in a variety of practice settings and a textbook (Asen and Scholz 2010).

**Table 1:** Participating genetic counsellor’s outline demographic information

Name	Ethnicity	Qualifications related to genetic counselling	Genetic counselling experience (years)
Katie	White British	MSc in Genetic Counselling; PhD	5
Anna	White British	Post-Grad Dip in Genetic Counselling	11
Chandni	Bangladeshi	BSc in Medical Genetics, MSc in Ethics of genetics and MSc in Genetic Counselling	6

Genetic counsellors also participated in four informal training sessions, in which a family therapist (SH), the Principle Investigator (AM) and a researcher (ER) were present. Training focused on providing advice and guidance to GCs to address their concerns about the intervention, its delivery via their facilitation role and practicing different MFDG activities outlined in the textbook and training manual. Additionally, in April 2014 a mock MFDG was conducted. Families who had attended the focus groups in phase 1 were invited back to experience what had been developed from their discussions, to gain their feedback and support the GCs in their training. Furthermore, in May 2014 two GCs observed two MFDG sessions led by family therapists, with families affected by eating disorders. The third GC was unable to attend the sessions due to relocating to a different genetics centre. Finally, in July 2014 all three GCs conducted a MFDG role play session with their peers in the genetics centre, with one family therapist (SH) providing feedback on their performance.

Following the training sessions, the intervention was piloted over a weekend in November 2014 with a new cohort of six families, who had been recruited via advertisements on charities’ webpages, links with specialist genetics centres and the project’s postal survey. Information and findings about the survey are reported elsewhere (SPRinG collaborative 2015). The intervention was co-facilitated over two full days by the three GCs, family therapist (SH) and Principle Investigator (AM) and observed by one researcher (CJ) (See SPRinG, 2015).

To evaluate the training process three multi-perspective interviews (Kendall et al. 2009; Valentine 1999) were conducted with the three GCs together at three critical points; before, during and after the training. Interviews recorded the GCs’: shifting views, attitudes and beliefs about the value of the intervention for families, GCs and for the service provider, their experiences of the training process, their thoughts about whether GCs are the most appropriate health professionals to deliver this intervention and the implications of this intervention on the future delivery of genetic counselling services in the UK. The multi-perspective interviews lasted approximately 45–60 min and were conducted by one researcher (GR). Interviews were recorded using an encrypted recording device and transcribed verbatim and checked for accuracy by a researcher (ER).

### Data Analysis

Transcripts were inserted into ATLAS Ti 6.2.28 for data management and storage (Friese 2014). Data were analysed using thematic analysis (Boyatzis 1998; Braun and Clarke 2006; Fereday and Muir-Cochrane 2006; Joffe and Yardley 2004). Data were coded by two researchers (ER and CJ) applying inductive and deductive codes at a semantic level (Boyatzis 1998; Braun and Clarke 2006; Fereday and Muir-Cochrane 2006). Examples of deductive codes include: care politics, challenges of embedding MFDG into practice, current NHS climate, feasibility and implications to future practice. Line by line and lumper coding techniques were applied to the data (Fereday and Muir-Cochrane 2006; Saldana 2013). Following two rounds of coding, 10 % of data were checked by a third researcher (GR). To ensure consistent and rigorous coding practices, a “code manual” including a code list and code definitions was created (Saldana 2013). Discrepancies in code names and definitions were discussed amongst two researchers (ER and CJ) and adjusted accordingly. Established codes were collapsed and merged into code hierarchies and then translated into themes (Boyatzis 1998; Braun and Clarke 2006; Fereday and Muir-Cochrane 2006), queries were run in ATLAS Ti 6.2.28 to retrieve data for dissemination (Friese 2014). Pseudonyms are used for the quotes to protect the identity of the participants.

## Results

Thematic analysis established 5 themes and corresponding sub-themes. The five themes included: motivations for taking part in the research, perceived benefits of implementing MFDG, perceived challenges in implementing MFDGs, training GCs and piloting the intervention.

### Motivations for Taking Part

The reasons why the 3 GCs volunteered to participate in the research were divided into two sub-themes: professional development and supporting families.

#### Professional Development

The GCs believed that the research was novel and that the intervention could potentially transform the way in which genetic counselling services are delivered. They therefore wanted to be part of something exciting and innovative.

*Katie: …It seemed like a new skill and it…hasn’t been done before, so I* [would] *have something quite useful particularly if it got rolled out…*

*Anna: Similar for me,* [the research] *sounded quite exciting…something completely new…I also have a lot of concern about families communicating genetic results…so I want to know how to do it better and I thought I could learn a lot from this study (Multi-perspective interview 1)*

Genetic counsellors were motivated by the need to develop new skills. They were particularly keen to build their confidence in communicating with families and enhancing their knowledge and understanding of families’ physical and emotional needs, so that they would appreciate “*how hard it is for families to disclose genetic risk information*” (*Chandni, Multi-perspective interview 1*). Greater understanding would enable them to be more empathic towards their patients.

The GCs felt that the greatest proficiencies to be gained from the training would be in managing group dynamics and therapeutic counselling skills. The GCs stated that current training predominately focuses on individualised care directed towards the family member affected or at risk from the IGC; nonetheless, they appreciated that genetic risk information had wider family implications and therefore developing skills in family focused care would be highly beneficial. Katie and Anna both commented that their genetic counselling training had never exposed them to the idea of large family group counselling….

*Katie: …It’s part of our training as genetic counsellors to have some level of training in terms of communicating with families, but nothing in terms of where we are going with this.*

*Anna: …no family group therapy…*

*Katie: …In a typical session we would see either a patient on their own or a patient with their husband or wife…but rarely do you get more than three or four people coming into clinic… (Multi-perspective interview 1)*

Genetic counsellors were also keen to support families in more ways than providing genetic education, which can often be overwhelming for patients.

*Chandni: I think it would be nice to have some therapeutic skills because a lot of what we do is just information giving and sometimes you wish there was more counselling aspect to it because sometimes I feel like I talk too much because there is so much to get across… (Multiple perspective interview 1)*

#### Supporting Families

In addition to enhancing their own professional skills, GCs wanted to better support families affected or at risk from IGCs. They believed that the MFDG would benefit patients as current practice has limited opportunity for patient follow-up after diagnosis. The MFDG would provide families with advice and guidance in communicating genetic risk information to their family members, thus enhancing families’ functioning and emotional well-being.

*Chandni: …A lot of the time we don’t follow things up…we don’t have time…it’s to do with how much information we’re giving rather than therapeutic counselling, so this is a very good way of making sure we support them. (Multi-perspective interview 1)*

### Perceived Benefits of Implementing MFDG

Keen to be involved in the research, the GCs identified several potential benefits of the MFDG for GCS and families affected by IGCs. These benefits were divided into two sub-themes: Empowering families and families’ understanding the GC role.

#### Empowering Families

The main benefit of the MFDG would be to empower families, giving them the confidence to commence and continue conversations with their children and strengthen existing family bonds through discussions with other families in a safe and supportive environment. The most empowering aspect of the MFDG was perceived to be the way in which families would be facilitated to help themselves and support each other through their own lived experiences. Additionally, the MFDG would provide families with the skills to access their own information and establish supportive networks that could then be sustained outside of the MFDG setting.

#### Families’ Understanding Genetic Counsellors’ Role

In addition to empowering families, GCs felt that the MFDG would allow families to have a better understanding of their role. This was important to GCs because after attending the focus groups with families to co-design the MFDG intervention, they felt that families misunderstood their role, perceiving them to have a more therapeutic role than they currently provide (SPRinG collaborative 2015). GCs therefore wanted to be able to manage families’ expectations of service provision.

*Chandni: I was surprised by some of their expectations…one of them said ‘I would like genetic counsellors to be therapeutic counsellors.’ That is not what we do and I don’t think that’s going to change in the near future. We’re never going to be therapeutic counsellors.*

*Anna: We have too much information to give.*

*Chandni: Yeah, it’s very much focussed on genetics…we do want to learn the skills that help us help families, in terms of therapeutic interventions, but we are never going to be therapeutic counsellors. (Multi-perspective interview 2)*

Whilst recognising the potential benefits of the MFDGs, the GCs also perceived several challenges.

Perceived challenges in implementing MFDGs

Four challenges were outlined;

i) *Bringing multiple families together*

The GCs were candid in disclosing how the thought of bringing several families together was a source of anxiety for them; it was very different from current practice in which patients were given information individually. The MFDG group format was therefore considered “*out of [their] comfort zone” (Multiple-perspective interview 1)*. The GCs were particularly concerned about managing group dynamics and being able to sufficiently address multiple perspectives and / or individual family needs. Managing “difficult” or challenging families or family members was also a concern.

*Anna: …I worry about bringing all these families together…so many variants in the room, and it’s just the unknown…you don’t know what kind of personality you’re going to get and whether they could say damaging things to each other, and how do you mediate that? It’s a bit scary. (Multi-perspective interview 1)*

Initial concerns and anxieties about managing group dynamics were exacerbated by the mock MFDG where the GCs felt that the attending participants had been very articulate and empowered; atypical of the families they had experienced in clinic.

*Katie: …Having seen the mock family group and how it works… I’m so much more daunted by family dynamics…the kind of people who may come along and support this kind of intervention…families who are in some way campaigning or leading the way don’t generally have a good impression of genetic counsellors. (Multi-perspective interview 2)*

Furthermore, GCs were anxious that parents would bring children who had not received any genetic risk information, expecting that the GCs disclose information to them.

*Katie: I think this is what I am most frightened of…I guess we try to identify families that aren’t communicating and the ways in which we can potentially assist with that…I think my main fear…I still don’t have a feeling about how we are going to police or stop families from bringing their children to be told something, and that’s not what this is about. It’s not our job to sit down and tell their child about the family history… (Multi-perspective interview 1)*

Other challenges, centred on the logistical challenge of getting families together for a designated amount of time in spite of school and extra-curricular activities.

*Anna: I don’t really understand how you get all these families and professionals together at the same time every two weeks…even getting them for one day seems a huge challenge. (Multiple-perspective interview 2)*

#### Usefulness to Families

In addition to the challenges of bringing multiple families together, GCs were also aware that the MFDG needed to be useful to families. Making sure that the MFDG was beneficial to all families was challenging due to individual family (member) needs.

*Anna: My main concern is that if…we actually do it in the clinic and it doesn’t help everybody, or they don’t think that it’s as beneficial as we thought…because as you know every family is different, what might work for some, might not work for others. (Multi-perspective interview 1)*

ii) Recruitment and selection bias

Genetic counsellors suggested that recruitment, in particular selection bias, could affect the suitability and effectiveness of the intervention. They believed that families likely to participate in the research and in future MFDGs, may be those that are already highly motivated to communicate with their children, are articulate and already members of support groups. Families struggling to communicate may therefore not participate, creating a distorted view of the value and benefit of the MFDG to families affected by IGCs.

*Anna: I guess there will definitely be selection bias, it will probably be the more practiced families that want to improve and are motivated to make sure they do everything right, whereas there’s a lot of awful communication out there, people telling disastrous things to their kids, or not saying anything – heads in the sand….*

*GR: So you’re not going to get to those hard to reach kind of families.*

*Chandni: At the [focus] group we attended…the parents…were very articulate…they definitely [didn’t] represent the average patient…*

*Katie: But I guess you’re not trying to help everybody…there are a group of people who desperately want to improve the communication in their family but just don’t know how, or don’t have the confidence to, so it is about making a difference to those families who…have said, I need help… (Multi-perspective interview 1)*

#### Feasibility of Embedding the Intervention into Routine Service Provision

The most significant challenge to implementing the MFDG into clinical practice was the wider publically-funded healthcare context in which the genetics centre was situated in the UK. This context included increasing patient numbers and the external demands placed on GCs to see patients in a timely fashion.

*Chandni: A barrier to this intervention…is the environment that we are working in now. I mean we’re all absolutely stretched, we’re being asked to see more and more patients in less and less time, and although we’re all…highly motivated, even finding the time to…do this training is difficult, and so I think this poses a huge challenge for…future care. (Multi-perspective interview 2)*

In addition, wider financial constraints affecting the genetics centre made the implementation of a MFDG - as a service provision outside of “standard” care – challenging and difficult to justify.

The GCs thought that to reconcile the cost of implementing the intervention, the economic value of the MFDG would need to be demonstrated.

To implement this “additional service,” the GCs suggest that they would have to deliver it in their own free time, similar to a breast cancer support group which is currently organised from their genetics centre.

*Katie: …It might just mean that we’d have to give up our time, a lot like how the support groups are run for the BRCA breast cancer patients. They [run] them during the weekends and after work so people [GCs] volunteer to do it as extra time…outside of their work. (Multi-perspective interview 1)*

The fact that the GCs were considering conducting MFDGs in their own time, demonstrates their belief that it is “critical and important that [MFDGs] are available to families” (Multi-perspective interview 1). It was however not only the GCs participating in the research who perceived the benefit of the MFDG to families. The GCs reflected on how supportive not only their line manager and peers had been within their genetics centre, but also how positive and encouraging GCs from other genetic centres had been about the intervention.

*GR: Do you think there will be tensions within your professional group…around focusing on this type of intervention?*

*Chandni: I don’t think so at all…most people I’ve spoken to…are very positive…*

*Anna: I think certainly in our department the geneticists and the genetic counsellors, I think everyone would be quite encouraging…everyone we’ve spoken to…thinks it’s a great idea.*

*Chandni: I think everyone’s quite interested…nationally as well…genetic counsellors are always excited about something that might add to what they do. (Multiple-perspective interview 1).*

iii) **Training Genetic Counsellors**

Throughout the interviews the GCs reflected on their experiences of the training process. Discussions focused around the two aspects of training: the training manual and the “hands on” training activities.

#### Training Manual

The training manual contained literature and introductory text about the use of MFDG in practice and a textbook. In the first multi-perspective interview, the GCs expressed disappointment with the manual because they felt that it did not contain information about how the MFDGs would be structured and facilitated. It therefore did not answer any of the questions that they had surrounding the practicalities of the sessions.

*GR: Have you had a chance to flick through it [manual]…what were your first impressions?*

*Katie: …It didn’t really tell me anything, all of the background stuff…we were already familiar with, because we’ve been to lots of events about this…, but we hadn’t heard yet was how it would be structured…*

*GR: So you were left with pretty much the same questions as you had at the start?*

*Chandni: Some of it was quite interesting…but I was looking forward to learning about how the sessions are actually going to be set up… (Multi-perspective interview 1)*

By the time of the second multi-perspective interview the GCs had had more time to read the manual and the accompanying textbook, and whilst they still felt that it lacked vital information about the practical implementation of the MFDG they were more positive about the value of the manual to their training.

*GR: Have you now used the manual? What are your impressions of it over the last couple of months?…*

*Chandni: Yeah it’s really comprehensive. I think we’ve looked though all the different exercises and the readings.*

*Katie: I thought it was helpful. I think when I initially read it I thought that I’d like to see this in practice. I’m still not entirely sure how this is beneficial, but I understand what they are trying to do, and it makes a lot of sense. The book was also good; again it’s just seeing it in practice. (Multi-perspective interview 2)*

The manual did contain limited information about the logistics and practical application of the intervention. This was because the MFDG was being co-designed throughout the training process. Adapting the MFDG to be acceptable to families affected by IGCs was a learning process as much for the research team as well as for the GCs.

#### “Hands on” MFDG Training

Genetic counsellors were initially enthusiastic about the training and the new skills that they would develop. However, by the time of the second interview the GCs disclosed their disappointment in the training that they had received.

*GR: You have had two training sessions now, is that right?*

*Anna: Yes there were two*

*Katie: I wouldn’t define them as that…we have had two meetings, where we’ve talked about our concerns, but they haven’t felt very much like training. Is that fair?…*

*Chandni: I don’t know what I had in my head what the training would involve, but some kind of passing of knowledge or some kind of practicing or rehearsing. As in ‘this is how you do something, now let’s practice that.’ It hasn’t been that at all… (Multi-perspective interview 2)*

Training discussions were broken down into four different components: four training sessions; mock MFDG; observations of an MFDG in practice; and conducting MFDG activities with their peers. Below the GCs reflect on each of these components in terms of their overall training.

#### Training Sessions

The four training sessions were conducted by family therapist, SH and AM, and observed by researcher, ER. In the second interview the GCs described the content of the first two training sessions:

*GR: So what I had thought were the two sessions of training were in fact two meetings…Is that right?*

*Chandni: …We Asked them to Do an Exercise on us, so that we could Get an Idea and Stop them and Ask them What they Were Doing. So that Was Helpful*

*Katie: We didn’t have a lot of time for that though, did we? It wasn’t completely done, but it was probably the most helpful out of the two training days. (Multi-perspective interview 2)*

Following the fourth training session, the GCs were still not convinced that they had received formal training.

*Anna: I still think…looking back it didn't feel like formal training…Going into the [pilot intervention], I'm not sure it felt like I'd learned A, B and C, and that we'd had a training session that had covered that. (Multiple-perspective interview 3)*

Genetic counsellors believed that learning MFDG skills could only be achieved through practice.

#### Mock MFDG

During the mock MFDG the majority of the MFDG activities were facilitated and led by a family therapists SH, IE and AM, with the three GCs observing and providing support. Whilst not facilitating all of the activities, the GCs felt that the mock MFDG had been beneficial to their training as it had enabled them to observe a MFDG in practice and gain an understanding of what their role would be in the future.

Despite being a positive experience, the mock MFDG created anxieties as the GCs remained concerned about their ability to lead the intervention due to families’ agendas.

*Chandni: …I think the mock [MFDG] had still been set up very much as - it was all research, but this is how to design research, asking more questions about how we do things, would you please help us? It was very much people who wanted to get their own agenda talked about. They wanted to come and talk about how bad the service they'd experienced had been, and that under ran the whole day, and there were lots of negative connotations about all of that throughout the day which kind of simmered along. (Multiple-perspective 3)*

#### Observing an MFDG

The two attending GCs found the observing a MFDG over 2 days an enlightening experience, which supported their understanding and training needs. Despite finding it useful, the GCs also continued to reflect on the temporal and logistical challenges of being able to accommodate this element of the training around practice.

*Chandni: What was really helpful too, is that Anna and I went and observed an MFDG…seeing that first hand gave us a huge…confidence. (Multi-perspective interview 2)*

#### Practising MFDG Activities with Peers

During the training process the GCs wanted to practice the skills they had learnt with their genetic counsellor peers. However, finding the time within clinic to do so proved difficult.

*GR: [ER] did mention to me that you were thinking of doing a…session with your colleagues. It sounds like this hasn’t happened yet...*

*Anna: Everyone is just overloaded with work at the moment.*

*Katie: We’ve got a colleague off long-term sick, who kind of holds the whole department together, so it is all a bit crazy.*

*Anna: And everyone is having to cover her…so I’m not entirely sure whether we can ask them to do more work with us at this point.*

*Katie: Maybe we could ask people if they would do it in their own time… (Multi-perspective interview 2).*

By the third and final interview the GCs had managed to practice some of the MFDG activities with their colleagues and gather feedback from one of the family therapists (SH). The GCs found this practice session useful in building their confidence in the delivery of the intervention.

*Katie: [One day] we had [SH] with us, we did a kind of practice run of one of the exercises with some of our colleagues…I think it gave us a bit of confidence that yes, we can do this and it would be different [from the MFDG mock]. That was really helpful. (Multi-perspective interview 3)*

### Delivering the ‘Complete’ MFDG Intervention

In discussing the piloted, complete MFDG, which the GCs co-facilitated, four sub-themes emerged;

#### Implementation of the Intervention

All GCs were very positive about their experience of co-facilitating the pilot intervention and believed that the MFDG had been executed well, with both themselves and the families benefiting from the two day event.

*GR: When I spoke to you in May you'd just done the mock MFDG and there had been some issues there about how that session had gone…the kind of words you were using were 'daunting,' 'frightened,' 'worried about,'…My sense from [ER] and [AM] was that [the pilot intervention] went much better. So are you still feeling daunt[ed]…traumatized, or have things changed? [laughter]*

*Anna: No longer feeling traumatized, I have to say.*

*Katie: I think the fact that it's in the past now, it's not hanging over us, so definitely not feeling stressed....Immediately after* [the pilot] *I was on a real high*

*GR: So you were still anxious going into this real event …*

*Katie: Very anxious…I think we all weren't sure that it was really going to work, whether it would be beneficial to the families. I think by the end of it we all agreed that they did get something out of it, and we got something out of it as well, and it felt a lot…smoother…just seemed like it flowed better, and we were much…more confident in what we were doing…*

*Chandni: Yeah, I agree. I think going into it…[I was] wondering what it would be like and how it would work out. And yeah, I think we all came out after both days feeling like we'd really achieved something, both for us and for the families. And it was a really positive experience. Yes, it completely changed my mind. (Multi-perspective interview 3)*

Part of the reason why the GCs had felt so positive when reflecting on the pilot was that they had been introduced from the beginning as co-facilitators. This allowed the families to view them as skilled facilitators rather than GCs in training; this had boosted their confidence, allowing them to establish a different relationship to these families than they had been able to during the mock MFDG.

*Katie: I felt like the families there believed that we were in control more than they did when we were observing [the mock MFDG]. And I think the fact that we started it off with the icebreaker, from then on they, I guess, had some level of respect rather than us just being these people that had no idea what we were doing. So I think it's just the way they might have perceived us gave us the confidence just to go, 'Okay, we're believable. Let's go with this.'*

*Anna: That's a really good point, actually…because in the mock we were introduced as people very much learning, whereas in the real thing [pilot intervention]… we were introduced as co-facilitators from day one and that we would be running it, weren't we?*

*Chandni: Yeah. I think they saw us as more professionals. (Multi-perspective interview 3)*

Coming out of this positive experience, the GCs reflected on what they had learnt from facilitating the pilot intervention.

#### Learning Experiences

The most prominent learning experience for GCs was that they could be successfully trained to deliver the intervention. However, they still did not feel they had the confidence, knowledge or experience to conduct the intervention (or train their peers in the MFDG) without ongoing support.

*Chandni: I'm not really sure if the aim was to see if genetic counsellors could lead these independently…or if we could run them in conjunction with someone like* [SH]. *I don't know. I think we could do them, but I think they'd be a different format.*

*Katie: Yeah, and I also think we'd need to do a few more with them before we started doing them by ourselves.*

*Anna: Definitely.*

*Katie: I wouldn't have enough confidence to train genetic counsellors to do it until I felt confident actually running a whole thing myself. (Multiple-perspective interview 3)*

Genetic counsellors also felt that they had gained skills in managing group dynamics and that they would be able to deliver greater family oriented care in the future. They perceived this to be an exciting and innovative new direction for GCs.

*GR: Do you feel you have had personal development, new skills?...*

*Chandni: Yeah, I look back at it [involvement in research] as a really worthwhile thing I did in work; other than my just normal clinic work, definitely the most important I've done last year. It was something different and opened my eyes to lots of things…*

*GR: And so what are the new skills you've learned? Can you put a finger on..?*

*Chandni: I guess it's a whole new way of counselling patients. It's just something very, very new. I've never come across anything like this before. And up and until we did this I didn't even know that that was a possibility, getting a group together and doing counselling for genetic patients in that way, or any patients, really. It's a completely new technique for me to learn.*

*Katie: It's given me more confidence working with the family groups in clinic and given me more ideas and more tools to, I guess, get different people's opinions or to suggest ways that they might work on things outside of clinic. So I think it's made me much more family focused, although that's probably not right because I was before, but better at doing that, I guess.*

*Anna: Same for me. When people talk about difficulty in talking to family members I bring it back to that [focus] group…and normalise their feelings and hopefully use that positively. And the other thing is, just a more generic skill, is just working with groups. That was what intimidated me a bit... How do you work with 20 different people in the room and different families and dynamics? So actually doing that - and we did, we bounced around the room with it, which we'd never done before... I'd like to get better at that. That's definitely a new skill. (Multi-perspective interview 3)*

Whilst providing the GCs with new skills and an innovative way of working with families, the GCs (and the research team) learnt that the implementation of the intervention into future practice will continue to pose challenges.

*GR: What about from your side …Is it [the MFDG] feasible?*

*Katie: No… I think people would want to get time off if we were doing [the MFDG], or time in lieu…because I don't think it's possible to do it on top of everything that we do.*

*Anna: Yeah, it's really draining.*

*Katie: And it's a lot of work. It means giving up a weekend, because we gave up our weekend which we didn't get back, and I know we were paid for the weekend, but it just meant that we then worked a 12-day week... (Multiple-perspective interview 3)*

Additionally, the GCs talked about the challenges of getting families to commit to the MFDG over multiple sessions, or fitting in with their work, school and extra-curricular activities.

*Chandni: “It really struck me how logistically difficult these are…we were trying to work around these families, 'When can we fit you in? School holidays are good,' 'No, they're bad.' It's just so hard. And then getting us in for a weekend as well. And even though it was brilliant as a one-off I couldn't work through a weekend every week, and I think it's a lot for parents to do that, too…” (Multi-perspective interview 3)*

Genetic counsellors also recognised that the MFDG template used with families suffering from anorexia or other conditions would not work with families with genetic conditions and therefore a MFDG schedule would need to be adapted specifically for the needs and acceptability of these families.

*Chandni: We'd always wanted to do it over four days…And then the families just wouldn't commit. So we went, 'Right, we'll cut it down to two days’; it's …about working out what works in genetics…*

*GR: And do the three of you have any sense of what might be best?*

*Katie: I think it's really difficult to say just because you wouldn't know how much intervention they would need or how much they'd consider to be too much, because it is a lot to ask them to come over for two whole days, let alone four days. Because remember…the MFDG…[observed] was anorexia, and it was teenagers whose parents were desperate; this is what they needed to do in order for them to survive. Whereas with the genetic conditions, probably not the same…* (Multi-perspective interview 3)

#### Patient Outcomes

In terms of MFDG outcomes the GCs stated that they had felt that it was an opportunity for children to communicate with their parents in a safe and open space. This had brought families together and allowed parents to realise that their children had a lot of insight into their family’s genetic condition. The MFDG had brought family members together who had not spoken in years and had resulted in communication channels being re-opened. Whilst some benefits had been obvious, they recognised that all families had their own challenges and reasons for attending the MFDG, and therefore it was quite difficult for them to pin down exactly what benefit the MFDG intervention had had on families or individual family members.

*GR: And what do you think you perception is of what the families got out of it?*

*Katie: …This is about getting parents to talk to their children about genetic disease. A lot of them were already talking. What I realised was that every single family had really complicated issues, some of them completely unrelated to each other. But whatever it was, they all got something out of it…There were family members who hadn't talked to each other in years and so many dynamics of just being together in that room.*

*Anna: ….When they [families] fed back they said how much they got out of it. I'm not entirely sure what it was that they got out of it. Obviously it helped them to talk about things but, at the same time - I'm not entirely sure, because, as you said, there were very different needs of different families. I'm not sure if we met all of them, but that probably wasn't the purpose...It was probably just to get them talking…or just them being in the same room… which I think was achieved… All the feedback was that they thought it was incredible. (Multi-perspective interview 3)*

#### Future Involvement

After reflecting on their training and pilot intervention the GCs discussed whether they would like to continue their participation in research to further develop and test the intervention for feasibility and acceptability in a future study.

*GR: Would you [want] any role in [future research]?...*

*Chandni: I think the plan is to have a role. I'm just not sure in what context…*

*Anna: …I feel a bit of a responsibility that we should be involved now that we've had all that training put into us. And I think that is the expectation. But I don't know in what capacity…*

*Katie: I would definitely want to be [involved]…talking about it, whatever …*

*GR: So you'd be advocates for the [intervention]…*

*Katie: Yeah. (Multi-perspective interview 3)*

The eagerness for the genetic counsellors to stay involved and engaged with the research indicates that they perceive value in the MFDG, for families affected and at risk of IGCs, their personal career development and for the future service provision of genetic counselling services. Their desire to stay involved in the research also indicates a positive experience in training and the research process.

## Discussion

Other studies designing and developing MFDGs have not attempted to train GCs in the delivery of the intervention. Instead family therapists have been used (Chiquelho et al. 2011; Mendes et al. 2010; Mendes et al. 2013). This makes this research project unique and innovative. It questions whether the role of GCs should be expanded from one that tends to be focused on information provision and facilitating decision making to a more psychotherapeutic role, assisting people in using the information and incorporating it into their family’s understanding and coping with the IGC, in line with accepted definitions of genetic counselling. There have been recent calls for greater uptake of psychotherapeutic approaches to genetic counselling practice (Austin et al. 2014).

The MFDG was perceived to be of value to GCs not only by the GCs involved in the research, but for GCs more widely. GCs saw the benefit of this intervention not only for themselves in terms of developing new skills, but also to families in facilitating family communication of genetic risk information and shifting the focus of care from individualised to family centred care and creating new supportive networks. In other health care settings MFDGs have been used successfully to support whole family’s needs (Asen and Schuff 2006; Eisler et al. 2007; McFarlane 2004; Mendes et al. 2015; Simic and Eisler 2015) and strengthen family bonds. In addition they have established extra-familial networks that provide additional support and advice to families long after the MFDG has taken place (Mendes et al. 2010).

The multi-perspective interviews demonstrate that the GCs’ perceptions of the MFDG and training shifted over time. Whilst enthusiastic about the new skills that the training could potentially provide them to support their current practice, the GCs were initially anxious about whether they could be successfully trained to deliver the MFDG. The greatest cause for concern for the GCs was their ability to manage group dynamics. The GCs had been perturbed by previous interactions with families for example during the focus groups (SPRinG collaborative 2015) where they had experienced animosity and some hostility towards them due to families’ previous negative experiences with GCs. Many families expressed frustration with being given information but then not supported to cope with it.

The families from the focus groups were regarded as more engaged and articulate than the families they would typically see in practice and their empowered approach had raised concerns amongst the GCs. However, as the training progressed the GCs began to take a stronger leadership role in the MFDG sessions (after observing a MFDG in practice and conducting sessions with their peers) which enabled their confidence to grow, and they were more assured in their ability to manage group dynamics effectively.

The GCs were concerned about their ability to deliver a therapeutic intervention as genetic counselling skills are centred on information giving rather than therapy. As the training progressed the GCs’ anxieties diminished as they began to realise that they could develop their skills in this area and deliver the MFDG effectively, in collaboration with the family therapist initially. However, in the longer-term, with appropriate additional training, practice and supervision, we anticipate that the GCs could facilitate the sessions themselves.

As innovations in genomic medicine increase, the skills and training of GCs will need to change and adapt accordingly (Battista et al. 2012; Sahhar et al. 2005; Weil 2002) because it is increasingly clear that simply providing information can create a huge burden for families who struggle to manage the IGC within their family. Training in the delivery of MFDG could provide GCs with the counselling and group management skills that will facilitate and assist families in the communication of genetic risk information as well as support their emotional and psychological needs. This will be valuable as more families come forward requesting genetic testing or are found to be affected or at risk from IGCs (Chiquelho et al. 2011; Mendes et al. 2015; Mendes et al. 2012).

Throughout the training process the research team realised that it was important to build the GCs confidence in their skills to facilitate families’ participation and to manage difficult group dynamics. The GCs’ focus on information giving meant that they had more anxieties and concerns about managing families’ emotions than the research team had anticipated. The training process therefore took longer than initially envisioned because although genetic counselling services regarded themselves as ‘family-focused,’ the GCs had only limited experience of working with the family system and the dynamics involved. These findings may also explain why some families feel quite angry towards genetic counsellors as a professional group where there is the misunderstanding of role, and the genetic information is given to the family without further assistance about how to use it and cope with it.

Practicing MFDG activities with peers was of great benefit not only in allowing the GCs to gain confidence and gather feedback from the family therapist but also in keeping their peers engaged and involved in the research. These elements should be incorporated into future GC training, perhaps using experiential and reciprocal peer group supervision techniques as described by Sexton et al. (2013). Despite the anxieties experienced throughout the training, delivering the complete MFDG intervention was an extremely positive experience for the GCs. In taking ownership and leadership of MFDG activities their confidence increased and they realised that they had developed the skills necessary to facilitate the intervention. Being introduced as co-facilitators also increased their confidence and caused the families to regard them as skilled professionals rather than as trainees which helped them take a more authoritative role. The way in which GCs are presented to the families is therefore an important consideration for future research.

With respect to anxieties surrounding training, the GCs also shared their concerns for the implementation of the MFDG into genetic counselling services. These concerns were consistent throughout the training process. Genetic counsellors were particularly concerned about the challenges of implementing the MFDG within the current publically-funded healthcare context in the UK, especially due to limited (financial and human) resources. Mendes et al. (2012) also discussed how a lack of human resources posed challenges to the implementation of MFDGs into practice and suggested that without additional funding the implementation of these interventions would be difficult. In addition, whilst seeing the benefit and value of the MFDG to families, GCs felt that the intervention would be regarded as a luxury rather than “standard” practice and therefore would be costly to the organisation. Chiquelho et al’s. (2011) study however demonstrated that the implementation of MFDG with cancer patients did not result in increased costs to the health service, but instead reduced costs by diminishing patients and their families doubts and anxieties related to illness, increased adherence to treatments, reduced stress, prevented additional appointments and / or other service use and prevented family dysfunction and development of psycho-emotional problems. Kai et al. (2009) also showed that when patients are provided with inadequate information and support as a result of inadequate communication, the financial costs to the health service are increased (Kai et al. 2009).

Any future work to test the MFDG effectiveness will measure the outcomes for families to see whether it increases their concordance with treatments and reduces their use of other services, a current serious concern raised by previous work (Metcalfe et al. 2011). Furthermore, successful implementation of MFDG for families affected by IGC is dependent on healthcare commissioners agreeing to integrate MFDGs into practice and service delivery, as GCs cannot be expected to deliver this intervention within their own time.

### Study Limitations

The GCs had more anxieties and worries about their skills and abilities to facilitate a MFDG than we originally anticipated. In retrospect this was not helped by us developing the MFDG intervention alongside the training of the GCs. Our intention originally had been to take a flexible approach and design the content of the MFDG with the GCs, which we did as part of their training sessions. Using the training manual which held the evidence of what was needed within the sessions and some of the exercises that might be used to facilitate families in developing the relevant skills. We hoped the manual would assist the GCs in using some flexibility to change the sessions if it became apparent that something was not working. However, it is clear that the GCs need more development in systemic family theory and learning family intervention skills as a basis on which to build specific MFDG skills. Having successfully completed the first full MFDG session, there is a distinct framework in which to develop GCs skills in facilitating MFDG.

Implementation of the MFDG intervention faced competing demands. The research team were eager to train the GCs to meet project milestones and deadlines. Whilst, centre leaders were very facilitative in supporting training, the GCs time was constrained by their existing workloads and additional human resource challenges in their centre which put limitations on their availability and flexibility to train. Finally, our research was limited by the small sample size, training of genetic counsellors from one genetics centre and the challenges in recruiting patients to the pilot intervention. Initially, the research team aimed to recruit participants to four MFDG sessions during the week, however families did not feel that taking time out of work or school was feasible (SPRinG collaborative 2015). The research team therefore modified the MFDG schedule to accommodate families by holding the MFDG over a weekend. However, these delays meant increased training time for the GCs and provided important learning for the research team.

### Practice Implications

Future genetic counselling services will require a shift in focus away from individual care to family centred care to support families to cope with IGCs. In light of a new focus towards family centred care, the GCs in the study were keen to develop their therapeutic counselling skills to better support families with IGCs, to communicate genetic risk information and support their family functioning.

Furthermore, the GCs were keen to develop their group management skills so that they could support multiple families during MFDG sessions. If GCs are trained to develop these skills they will be able to offer follow-up support to families, which often cannot be provided due to increased patient demands and a dearth of time and resources. We recognise there are financial implications for any health service, but equally there are health and socio-economic consequences for families who separate or become dysfunctional through lack of support in managing the genetic condition and its risks.

### Research Recommendations

The GCs despite their initial concerns, were able to co-facilitate the MFDG and they are likely to be able to lead on facilitating the MFDG in the future with further practice and support. However we will need to evaluate their progress when this work goes forward.

Finding ways to assimilate the MFDG into routine clinical practice emerged as the biggest issue. There needs to be sufficient staff to enable genetic counselling services to support families in using the information that GCs give them, otherwise there is a risk that genetic counselling and testing information is harming family relationships. Families preferred a weekend delivery of the intervention because they did not have to disclose to work or school about the genetic condition in their family, fearing stigmatisation. This underlines why MFDGs are likely to be so beneficial to families in developing their confidence to be more open about the IGC. Therefore health care managers, genetic counsellors and their educators will need to take this into account when designing services and educational programmes (i.e., the genetic counselling Masters degree that is required to practice). With the advent of a new seven-day NHS in the UK, this might provide better scope for UK GCs to deliver MFDG within work-time. Nevertheless, this is an issue that needs resolving and working with purchasers of health care services (commissioners) should be an important part of future studies.

## Conclusions

To our knowledge the SPRinG collaborative are the first to train GCs in delivery of an MFDG intervention. Our findings show that despite initial anxiety during the early stages the GCs were able to co-facilitate MFDG, which the GCs and the families found valuable and beneficial. The GCs learnt more about families, and how to facilitate their adaptation to living with the genetic condition, in addition to managing family and large group dynamics. Whilst for families, it was hugely beneficial meeting others in similar circumstances, sharing experiences and finding new ways to cope with the IGC so that it was less central to their family’s life. However, further work is now required to evaluate the effectiveness of the MFDG and to test and enhance the feasibility of successfully integrating them into routine clinical practice.

This intervention provides an exciting opportunity for GCs to adapt their care delivery to the shifting genetic landscape. The integration of MFDG into GC practice has the potential to provide a suitable and cost effective way of supporting families affected by or at risk from IGCs. The implementation of such interventions however relies on changes in both the organisation of clinical genetics services and the professional development of genetic counsellors through the incorporation of more psychotherapeutic counselling and family group management skills into their training.
